# Lupenone Protects Neuroblastoma SH-SY5y Cells Against Methamphetamine-Induced Apoptotic Cell Death via PI3K/Akt/mTOR Signaling Pathway

**DOI:** 10.3390/ijms21051617

**Published:** 2020-02-27

**Authors:** Hyun-Su Lee, Eun-Nam Kim, Gil-Saeng Jeong

**Affiliations:** College of Pharmacy, Keimyung University, Daegu 42601, Korea

**Keywords:** methamphetamine, neuroblastoma, PI3K, Akt, apoptosis

## Abstract

Methamphetamine (METH) is an addictive psychostimulant showing neurotoxicity through neuronal apoptosis and the neuro-inflammatory pathway. Lupenone, a lupane triterpenoid, is an isolated compound exhibiting anti-oxidative, anti-inflammation, and anti-diabetic activities. However, whether lupenone plays a protective role against apoptosis induced by METH in SH-SY5y neuroblastoma cells remains unknown. In the present study, we elucidated that lupenone had no toxicity to SH-SY5y cells at different concentrations. On the other hand, we found that the treatment of SH-SY5y cells with an optimal concentration of lupenone could lead to protection against cell death induced by METH. AnnexinV/PI apoptosis analysis revealed a dramatically reduced level of the apoptotic cell population in lupenon and METH treated SH-SY5y cells. Moreover, diminished expression of anti-apoptotic proteins, including Bcl-2, Caspase3, Caspase7, and Caspase8 in METH-exposed SH-SY5y cells, was significantly recovered by treatment with lupenone. This protection in the expression of anti-apoptotic proteins was due to an increased phosphorylation level of PI3K/Akt in METH-treated SH-SY5y cells pre-incubated with lupenone. These findings suggest that lupenone can protect SH-SY5y cells against METH-induced neuronal apoptosis through the PI3K/Akt pathway.

## 1. Introduction

Methamphetamine (METH) is an unlawful drug that causes illness and addiction. During recent decades, METH addiction has raised severe social issues for damaging public health in the United States. Several studies have reported that chronic METH administration can lead to symptoms of Parkinson’s disease, mainly due to neurotoxicity and neurodegeneration [1,2]. Although the underlying mechanism involved in dopaminergic neurotoxicity and neurodegeneration caused by METH remains unclear, the potential process can be inflammation, oxidative stress, and apoptosis [3,4,5]. Accumulated data have revealed that METH leads to inflammation and oxidative stress on neurons by producing pro-inflammatory molecules [5,6]. Since most of the inflammatory reactions and oxidative stress are mainly accompanied by neurotoxicity, cellular apoptosis of neurons by METH administration is one of the obvious manifestations of METH abuse [7,8,9,10]. Despite extensive efforts to develop pharmacological agents that attenuate neuronal toxicity occurred by METH administration, few pharmacological treatments for side effects of METH have been approved by the FDA [11]. Novel compounds that can regulate neuronal apoptosis and neurotoxicity might have the potential to be developed as therapeutics for preventing METH-induced neurodegeneration.

The PI3K/Akt/mTOR pathway is an intracellular signaling pathway that is required for cell cycle, proliferation, cell death, and autophagy [12,13,14]. It can be activated by various cellular stimuli and toxins [15]. Previous studies on PI3K inhibitors have demonstrated that PI3K can transduce a survival signal and result in the phosphorylation of Akt, one of the downstream molecules [16,17,18]. It has been elucidated that Akt can promote survival signals on growth factors by triggering the regulation of pro-apoptotic protein expression, another protein kinase, and transcription factors [19,20]. Mammalian target of rapamycin (mTOR) is also a well-known central regulator that controls cell death and autophagy against extrinsic stimuli such as growth factors, hormones, stress, and nutrients [21]. Since mTOR complex activates and phosphorylates Akt, PI3K/Akt/mTOR pathways are considered as a regulator of cell survival, cell death, and autophagy [22,23]. Bcl-2 family is one of the critical proteins involved in cellular survival controlled by the Akt pathway [24]. Several studies have reported that METH administration can dephosphorylate PI3K/Akt/mTOR [23,25] and suppress the expression of Bcl-2 on astrocytes [23]. Understanding the PI3K/Akt/mTOR pathway in METH-treated neuroblastoma cells is pivotal for establishing novel therapeutics against side effects of METH. Thus, it can be used as a strategy for the development of pharmacological agents targeting PI3K/Akt/mTOR pathways for the protection of neurotoxicity induced by METH.

Lupenone, one of lupane type triterpenoids, is a natural ingredient distributed in multiple plants, including *Asteraceae*, *Balanophoraceae*, *Iridaceae*, *Musaceae*, and *Bombacaceae* [26,27]. It has been discovered to have various activities, including anti-diabetic, anti-tumor, and anti-inflammatory activities [28,29,30]. In particular, lupenone can dramatically suppress NO production in LPS-stimulated RAW264.7 cells without any cytotoxicity [31]. Furthermore, in silico analysis to predict the biological role of lupenone has exhibited that lupenone is associated with PI3K/Akt and NFκB pathways [28]. However, although PI3K/Akt and NFκB pathways are known as apoptosis-associated pathways, whether lupenone has an anti-apoptotic effect against the death of dopaminergic neuroblastoma cells induced by METH has not been reported. The present study explored the anti-apoptotic role of lupenone on METH-induced cell death using SH-SY5y neuronal cells by regulating PI3K/Akt/mTOR pathways in vitro.

## 2. Results

### 2.1. Lupenone is not Cytotoxic to Neuroblastoma SH-SY5y Cells

**Figure 1:**
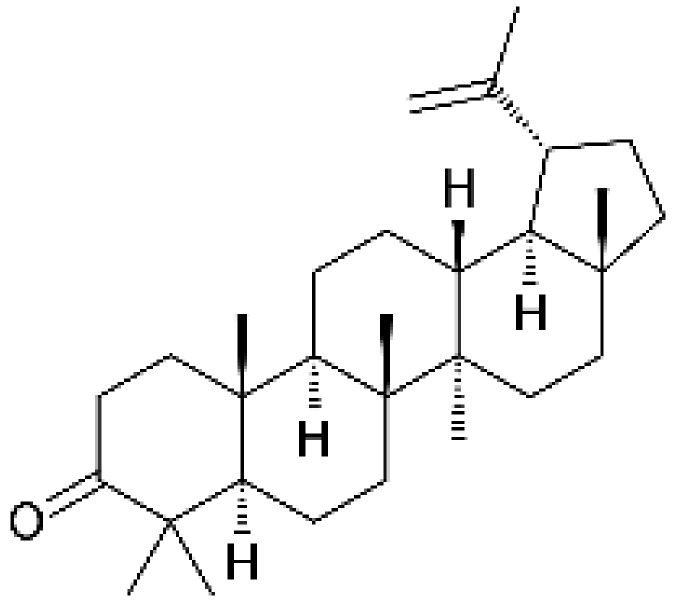
Chemical structure of lupenone.

**Figure 2:**
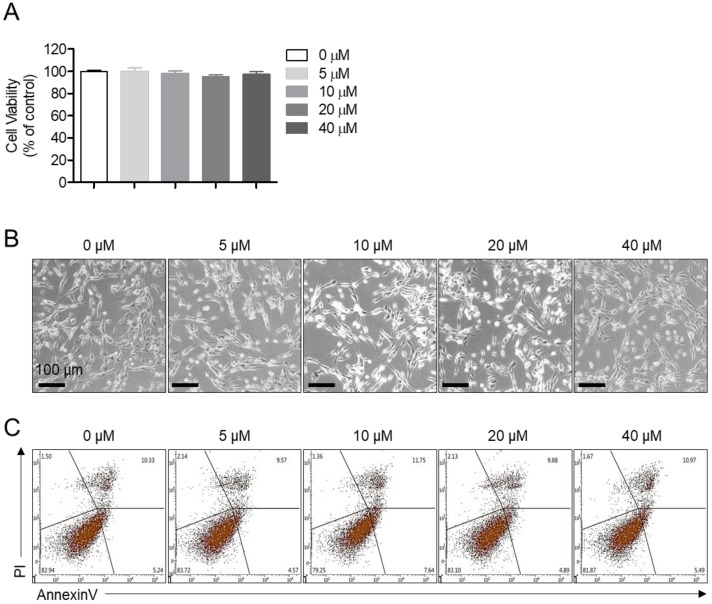
Lupenone is not cytotoxic to neuroblastoma SH-SY5y cells. (**A**) SH-SY5y cells (2 × 10^4^) were treated with the indicated concentration (5–40 μM) of lupenone in 96-well plates for 24 h, and viability was measured by MTT assay. (**B**) After treating SH-SY5y cells with lupenone (5–40 μM) for 24 h, images were taken with a bright-field microscope. (**C**) SH-SY5y cells (2 × 10^5^) were administrated with the indicated concentration (5–40 μM) of lupenone in 6-well plates for 24 h, and apoptotic cells were evaluated by annexinV/PI assay. Black bars in micrograph panels represent 100 μm. The mean value was calculated from the obtained data of three separate experiments.

We first determined whether lupenone (Figure 1) was cytotoxic to neuroblastoma SH-SY5y cells. MTT assay results demonstrated that lupenone did not cause significant cell death at different concentrations (Figure 2A). As shown in bright-field images containing SH-SY5y cells treated with the indicated concentration of lupenone for 24 h, lupenone did not affect the shape or the morphology of cells (Figure 2B). To investigate whether SH-SY5y cells might undergo apoptosis pathway after treatment with lupenone for 24 h, we performed an annexinV/PI staining assay. As shown in Figure 2C, lupenone did not induce annexinV-positive cells or annexinV/PI-positive cells, suggesting that lupenone was not involved in cell apoptosis of SH-SY5y cells.

### 2.2. Treatment of SH-SY5y Cells with Lupenone Blocks METH-Induced Cell Death

**Figure 3:**
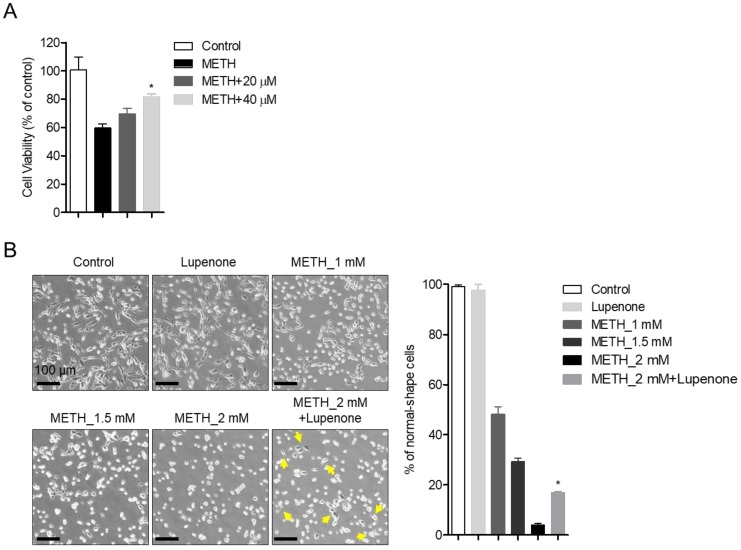
Treatment of SH-SY5y cells with lupenone blocks methamphetamine (METH)-induced cell death. (**A**) SH-SY5y cells (2 × 10^4^) were pre-treated with the indicated concentration (20 and 40 μM) of lupenone in 96-well plates for 1 h and stimulated with 2 mM of METH for 24 h. After incubation, cellular viability was measured by MTT assay. (**B**) Bright-field images of SH-SY5y neuroblastoma cells pre-treated with or without 40 μM of lupenone for 1 h followed by treatment with 1, 1.5, or 2 mM of METH for 24 h taken by a microscope. Cells showing regular or rescued morphology were counted for randomly taken images (at least three images per samples), and % of normal-shape cells are indicated in the graph (right). Black bars in micrograph panels represent 100 μm. Yellow arrows in cells treated with 2 mM METH and lupenone indicate SH-SY5y cells showing recovered morphology. The mean value was calculated from the obtained data of three separate experiments. *, *p* < 0.05, versus the METH group.

As we mentioned above, preventing METH-induced neurotoxicity is a critical strategy to develop drugs for neurological disorders, including Parkinson’s disease (PD) [31]. To explore whether lupenone could reduce dopaminergic neurotoxicity of METH to SH-SY5y neuroblastoma cells, MTT assay was performed with SH-SY5y cells pretreated with 20 or 40 μM of lupenone for 1 h followed by incubation with 2 mM of METH for 24 h (Figure 3A). Results demonstrated that the viability of SH-SY5y cells pre-treated with lupenone (20 and 40 μM) was elevated compared to that of control cells. Typical morphological changes, including shrinkages, membrane bleb formation, detachment from the surface, and aggregation, were observed in SH-SY5y cells after treatment with METH [32]. Consistent with MTT assay results, changes in shapes were monitored in METH-treated SH-SY5y cells in a dose-dependent manner. However, pre-treatment with lupenone rescued such morphology changes in METH treated SH-SY5y cells compared to cells treated by METH without pre-treatment with lupenone (Figure 3B). Accordingly, these data suggest that lupenone can effectively recover SH-SY5y neuroblastoma cells from METH-induced neurotoxicity in terms of cell viability and morphological changes.

### 2.3. Lupenone Protects SH-SY5y Cells Against METH-Induced Apoptosis

**Figure 4:**
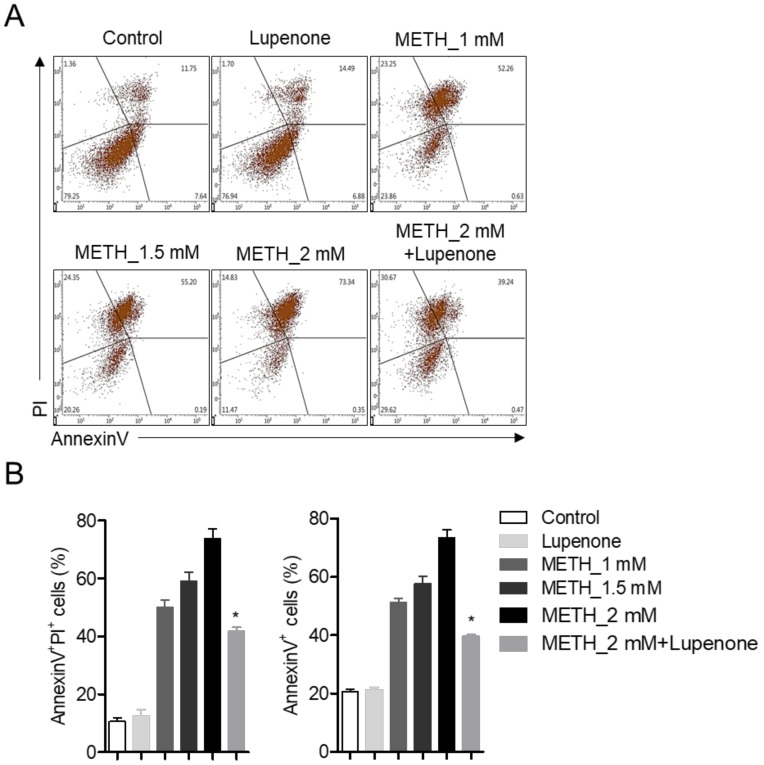
Lupenone protects SH-SY5y cells against METH-induced apoptosis. (**A**) SH-SY5y cells (2 × 10^5^) were pre-treated with or without 40 μM of lupenone in 6-well plates for 1 h and then administrated with 1, 1.5, or 2 mM of METH for 24 h. After incubation, cells were re-suspended with TE buffer and stained with annexinV conjugated with FITC and PI for 15 min at 37 °C. Stained cells were acquired by flow cytometry. (**B**) AnnexinV and PI-positive cells (late apoptotic cells, left) and annexinV positive cells (apoptotic cells, right) were included in the bar graph. The mean value was calculated from the obtained data of three separate experiments. *, *p* < 0.05, versus the METH_2 mM group.

The protection conferred by pre-treatment of lupenone against METH-induced apoptotic neurotoxicity was measured by flow cytometry analysis. AnnexinV staining with PI was performed with SHSY-5y cells treated with METH and 40 μM of lupenone. Dot plot results from flow cytometry analysis revealed that METH dramatically induced apoptotic SH-SY5y population within 24 h concentration-dependently. However, pre-treatment with lupenone reduced the population of annexinV/PI positive cells (early apoptotic cells) and annexinV positive cells (total apoptotic cells) (Figure 4A,B). These flow cytometric analysis results indicate that METH-induced neurotoxicity is associated with the apoptosis pathway and that lupenone can significantly diminish the apoptotic population induced by METH.

### 2.4. Lupenone Treatment Increases the Expression of Anti-Apoptotic Proteins After METH Stimulation in SH-SY5y Cells

**Figure 5:**
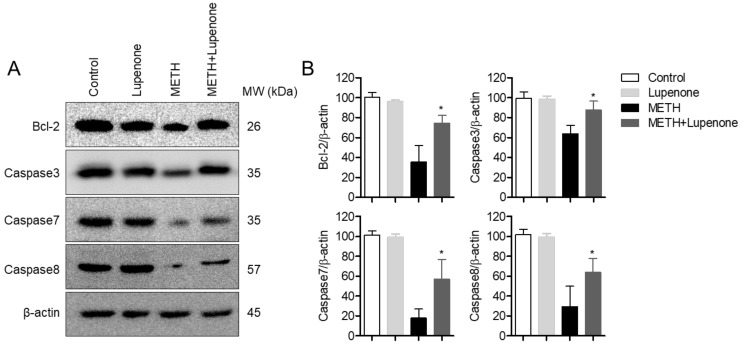
Lupenone treatment increases the expression of anti-apoptotic proteins after METH stimulation in SH-SY5y cells. (**A**) SH-SY5y cells (1 × 10^6^) were pre-incubated with 40 μM for 1 h and stimulated with 2 mM of METH for 24 h. After treatment, cells were harvested and lysed in RIPA buffer. Indicated protein expressions were detected by western blot analysis. (**B**) Densitometer results of western blot analysis from Figure 5A. Each band intensity was normalized to the band intensity of β-actin. *, *p* < 0.05, versus the METH group.

Cell apoptosis is controlled by many regulators, including the caspase family [33]. To investigate molecular changes in the expression of anti-apoptotic proteins, western blot analysis was performed with lysates from SH-SY5y cells with different incubation conditions. As shown in Figure 5A, the expression of Bcl-2, one of the anti-apoptotic proteins, was dramatically decreased in METH-treated SH-SY5y neuroblastoma cells but was rescued in lupenone pre-treated cells. On the other hand, the expression of caspase proteins, including caspase3, caspase7, and caspase8, were recovered in METH-treated SH-SY5y cells. These results suggest that treatment with lupenone can elevate the expression of anti-apoptotic proteins, including bcl-2 and caspases such as caspase3, caspase7, and caspase8 in METH-treated SH-SY5y neuroblastoma cells.

### 2.5. Lupenone Administration Recovers the Phosphorylation of PI3K/Akt/mTOR in METH-Treated SH-SY5y Cells

**Figure 6:**
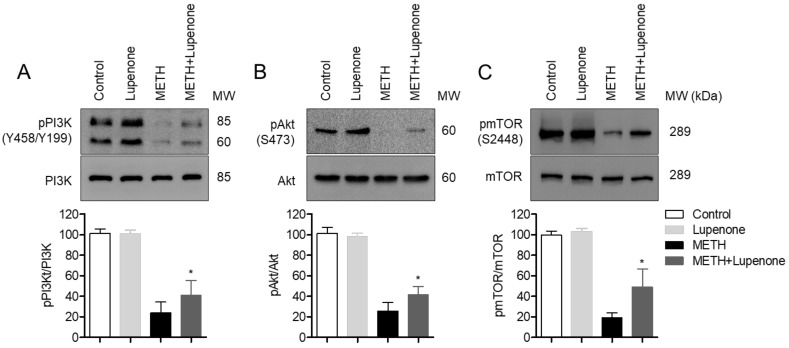
Lupenone administration recovers the phosphorylation of PI3K/Akt/mTOR in METH-treated SH-SY5y cells. (**A**–**C**) SH-SY5y cells (1 × 10^6^) were pre-incubated with 40 μM lupenone for 1 h and stimulated with 2 mM of METH for 1 h. After treatment, cells were collected and lysed in RIPA buffer. Levels of phosphorylated PI3K (**A**), phosphorylated Akt (**B**), and phosphorylated mTOR (**C**) expressions were detected by western blot analysis. Densitometer results of western blot analysis from upper figures. Each band intensity was normalized with the band intensity of PI3K (**A**), Akt (**B**), and mTOR (**C**), respectively. The detected phosphorylated PI3K sites were tyrosine458 and tyrosine199, the detected phosphorylated Akt site was serine473, and the detected phosphorylated mTOR site was serine2448. *, *p* < 0.05, versus the METH group.

The PI3K-Akt-mTOR pathway is known to be involved in the regulation of cell survival and apoptosis after treatment with METH [14,23]. Thus, we evaluated the phosphorylation level of PI3 kinase in SH-SY5y cells after METH treatment by western blot. PI3K phosphorylation level was diminished in METH-treated SH-SY5y cells but was recovered in lupenone pre-treated SH-SY5y before METH administration (Figure 6A). We further checked whether Akt, a downstream signal of PI3K, was affected by lupenone treatment. As we expected, pre-treatment with lupenone rescued the phosphorylation level of Akt in METH-treated SH-SY5y cells (Figure 6B). Several pieces of evidence have demonstrated that mTOR pathway also activates Akt phosphorylation in cells. We further explored if pre-treatment with lupenone recovered the activity of mTOR. These western blot analysis results suggest that neuronal apoptosis of SH-SY5y cells induced by METH treatment occurs through the PI3K-Akt pathway and that lupenone application to SH-SY5y before METH treatment can recuperate the phosphorylation level of PI3K and Akt.

### 2.6. Treatment with Lupenone Blocks Translocation of p65 and Degradation of IκBα

**Figure 7:**
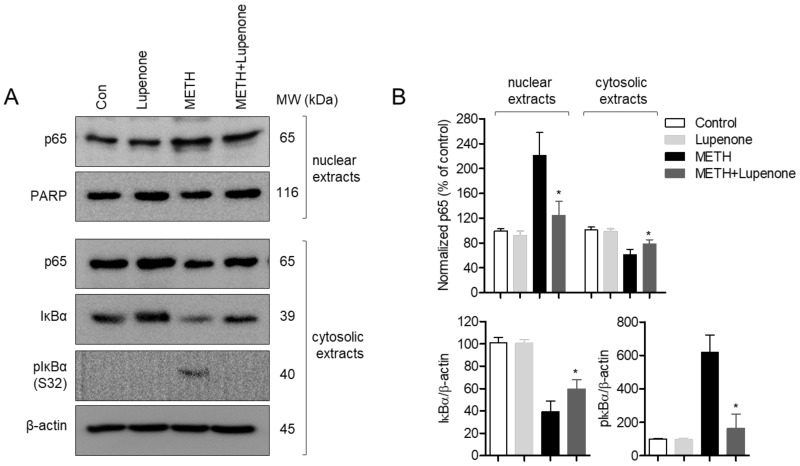
Treatment with lupenone blocks the translocation of p65 and degradation of IκBα. (**A**) SH-SY5y cells (1 × 10^6^) were pre-incubated with 40 μM lupenone for 1 h and stimulated with 2 mM of METH for 1 h. After treatment, cells were collected, and cytosolic extracts and nuclear extracts were separated using NE-PER Nuclear and Cytoplasmic Extraction Reagents. Indicated protein expressions were detected by western blot analysis. (**B**) Graphs demonstrate normalized expression of indicated proteins against the expression of PARP (for nuclear extracts) or β-actin (for cytosolic extracts), respectively. Detected phosphorylated IκBα site was serine32. *, *p* < 0.05, versus the METH group.

Various studies have demonstrated that translocation of p65, one of the subunits of NFκB, is the underlying mechanism involved in the effect of methamphetamine on neurotoxicity and neuroinflammation [34,35]. To explore the correlation between the action of lupenone in SH-SY5y cells and the NFκB signaling pathway, western blot analysis was performed with SH-SY5y cell lysates under different treatment conditions. As shown in Figure 7A, levels of p65 in the nuclear extract from SH-SY5y cells incubated with 2 mM of METH for 1 h were escalated, consistent with previous results [34,35]. However, treatment with lupenone decreased levels of p65 in nuclear extracts from METH-treated SH-SY5y cells. In contrast, administration with lupenone to METH-incubated SH-SY5y cells enhanced levels of p65 in cytosolic extracts. Degraded levels and phosphorylated levels of IκBα in cytosolic extracts from SH-SY5y cells were measured by western blot to determine whether lupenone was involved in the NFκB pathway. Consistently, METH-stimulated SH-SY5y cells revealed degraded IκBα as well as increased levels of phosphorylated IκBα. However, treatment with lupenone significantly counterbalanced the effects of METH administration on SH-SY5y neuroblastoma cells. These western blot data suggest that lupenone can impede effects caused by METH treatment, including the translocation of p65 from the cytosol to the nucleus, IκBα degradation, and IκBα phosphorylation in SH-SY5y cells.

## 3. Discussion

We explored whether METH administration could lead to neuronal apoptosis using SH-SY5y neuroblastoma cells. We also investigated whether lupenone, isolated from *Sorbus commixta*, might play a critical role in the attenuation of neuronal cell death induced by METH. We discovered that pre-treatment with lupenone of METH treated SH-SY5y cells significantly blocks the expression of Bcl-2 and Caspase family proteins, including -3, -7, -8. The rescued phosphorylation level of PI3K, Akt, and mTOR by pre-treatment with lupenone of METH treated SH-SY5y cells were confirmed. Furthermore, it was shown whether lupenone is involved in suppression of p65 translocation and IκBα degradation occurred by METH administration. These data suggested whether lupenone has a protective role in neuronal apoptosis induced by METH through the recovery of anti-apoptotic protein expression, the phosphorylation level of PI3K/Akt/mTOR pathways, and NFκB pathways.

In this study, we evaluated the protective effect of lupenone on neuronal cell death induced by METH using SH-SY5y neuroblastoma cell lines. SH-SY5y cells are human neuroblastoma that possesses many features of dopaminergic neurons, including the expression of tyrosine hydroxylase and dopamine transporter [36]. This in vitro system is widely used to investigate neuroprotection, neurotoxicity including cell death induced by METH [35,37,38,39] and neurodegenerative diseases like Parkinson’s disease (PD) since the loss of neuronal cells including dopaminergic neurons characterizes PD. In spite of many of these advantages, this system using SH-SY5y cells has limitations for PD and neurotoxicity study since SH-SY5y cells were originated from cancerous neuroblastoma and have undergone clonal changes through three rounds of clonal selection [40]. Besides, previous reports have been shown that differentiation of SH-SY5y cells using retinoic acid is beneficial for reflecting similarity with primary neurons that differentiated SH-SY5y cells obtain homogeneity, long branched processes, and express several markers of mature neurons [41,42]. In the present study, our data demonstrated the effects of lupenone on neurotoxicity induced by METH with molecular mechanisms using undifferentiated SH-SY5y neuroblastoma cell lines. To overcome the limitation of cell line systems, further studies are needed to confirm the underlying mechanism of how lupenone affects neuronal cytotoxicity with differentiated SH-SY5y cells and primary neurons and to administrate lupenone to PD animal models for obtaining therapeutic potentials.

The PI3K/Akt pathway is known to be involved in cellular homeostasis, including proliferation, development, and apoptosis [12]. For retaining biological equilibrium, cell death should be well-programmed and tightly controlled by anti-apoptotic and pro-apoptotic proteins. One of the pivotal roles of PI3K/Akt pathway in terms of apoptosis is that it can manage the expression of Bcl-2 protein family members, including Bcl-2, Bad, Bax, and caspase family proteins in stimulated condition. By modulating the balance between anti-apoptotic and pro-apoptotic proteins, the PI3K/Akt pathway decides cellular fate depending on the stimulation condition. Efforts to find target proteins of lupenone pharmacologically were recently reported by Wu and his colleagues in 2019 [28]. Functional enrichment analysis with candidate target genes was performed according to the functions of candidate genes by KEGG (Kyoto Encyclopedia of Genes and Genomes) pathway. Their results suggested that lupenone has a high possibility to be involved in apoptosis, osteoclast differentiation, NFκB signaling, and PI3K/Akt signaling pathway [28]. In this study, we demonstrated that treatment with lupenone upregulated the expression of Bcl-2 and caspases family (-3, -7, and -8) in METH-exposed condition. Phosphorylated levels of PI3K/Akt pathways were rescued in METH treated SH-SY5y cells that were pre-treated with lupenone before METH treatment. Besides, p65 translocation and IkBa degradation were mitigated in METH-stimulated SH-SY5y cells by pre-treatment with lupenone. Therefore, previous pieces of evidence predicting the target of lupenone in silico are consistent with our data that might be the first biochemical evidence to show the cellular effects of lupenone in terms of neuronal cytotoxicity induced by METH treatment.

Autophagy is a highly controlled molecular process, including recycling or degradation of cellular resources for cell survival and cell proliferation [43]. Endoplasmic reticulum (ER)-stress is one of the critical stimuli that lead cells to undergo autophagy [44,45]. In recent years, accumulating reports demonstrated that METH exposure induces ER-stress, which occurs neuronal cytotoxicity, including apoptosis and autophagy, simultaneously [46,47]. Especially PI3K/Akt/mTOR signaling pathways are known as pivotal regulators of apoptosis and autophagy induced by METH treatment [23]. On the other hand, several studies have demonstrated that ER-stress induced by METH also contributes to the increment of mitogen-activated protein kinase (MAPK) pathway, including ERK, JNK, and p38 [35,48,49]. Interestingly, crosstalk between PI3K/Akt/mTOR pathways and MAPK pathways has been investigated that elevated ERK negatively affects mTOR phosphorylation through TSC1-TSC2 complex and Rheb [50]. In the current studies, we have shown that METH treatment with SH-SY5y neuroblastoma cells reduces the phosphorylation level of PI3K, Akt, and mTOR but pre-treatment with lupenone partially recovers. These improvements suggest that not only lupenone attenuates PI3K/Akt/mTOR pathway to protect cells from neurotoxicity, but it is also possible that it blocks the MAPK pathway, including ERK in METH exposure condition. Further studies are needed if lupenone plays an inhibitory role in ERK phosphorylation induced by METH to address which signaling pathways are mainly involved in protection from neurotoxicity.

## 4. Materials and Methods

### 4.1. Cell Culture

SH-SY5y neuroblastoma cells (ATCC CRL-2266, Manassas, VA, USA) were purchased from the Korean Cell Line Bank (Seoul, Korea). The cells were identified with STR profiling, including D5S818, D13S317, D7S820, vWA, FGA, TH01, TPOX by Korean Cell Line Bank before distribution. The cells were cultured in DMEM medium (Welgene, Gyeongsan-si, Korea) supplemented with 10% fetal bovine serum (FBS), penicillin G (100 units/mL), streptomycin (100 μg/mL), and l-glutamine (2 mM), and grown at 37 °C in a humidified incubator containing 5% CO_2_ and 95% air. Cells were maintained within passage #15 prior to any experiments.

### 4.2. Lupenone Extraction and Isolation

Extraction of lupenone from the dried stem bark of *S. commixta* (1 kg) was performed with MeOH (2 L) by reflux for 2 h at 85 °C and filtered. The MeOH extract (121 g) was suspended in H_2_O (1 L) and then partitioned with *n*-hexane (1 L), EtOAc (1 L) and *n*-butanol (1 L). Among them, the EtOAc fraction (21.4 g) was open column chromatographed on silica gel (6.5 × 60 cm; 70–230 mesh) using a gradient of *n*-hexane−CH_2_Cl_2_ (1:3 *v*/*v*) to obtain Fr. 1–9. Of these, Fr. 5 (4.6 g) was eluted with *n*-hexane-acetone (10:2 *v*/*v*) on a Sephadex LH-20 column chromatography and purified by a silica gel column eluting with *n*-hexane-MeOH (4:1 *v*/*v*) to yield Fr. 5–1. The Fr. 5–1 was analyzed by ^1^H, and ^13^C-NMR (JEOL JNM-ECA 500) NMR spectroscopic, and spectral data were identified as lupenone (24 mg) by comparison with the reported literature [26]. Lupenone: ^1^H-NMR (500 MHz, CDCl_3_) δ: 4.70 (1H, m, H-29α), 4.55 (1H, m, H-29β), 2.51 (1H, m), 1.88 (2H, m), 1.65 (3H, s), 1.03 (3H, s), 0.99 (3H, s), 0.94 (3H, s), 0.77 (3H, s). ^13^C-NMR (500 MHz, CDCl_3_) δ: 54.4 (C-5), 49.5 (C-9), 49.2 (C-18), 48.0 (C-19), 47.1 (C-4), 43.1 (C-14), 43.0 (C-17), 40.7 (C-8), 40.0 (C-22), 39.7 (C-1), 38.3 (C-13), 37.0 (C-10), 34.2 (C-2), 35.7 (C-16), 33.8 (C-7), 29.9 (C-21), 27.3 (C-15), 27.0 (C-23), 25.1 (C-12), 21.5 (C-11), 21.3 (C-24), 19.7 (C-6), 19.4 (C-30), 18.4 (C-28), 16.2 (C-25), 16.0 (C-26), and 14.8 (C-27).

### 4.3. Reagent and Antibodies

Anti-Bcl2 and anti-phosphorylated mTOR (S2448) antibodies were obtained from Santa Cruz Biotechnology (Dallas, TX, USA). Anti-Caspase3, anti-Caspase7, anti-Caspase8, anti-β-actin, anti-phosphorylated PI3K (Y458/Y199), anti-phosphorylated Akt (S473), anti-p65, anti-PARP, anti-IκBα, anti-phosphorylated IκBα (S32), and anti-mTOR antibodies were purchased from Cell Signaling Technology (Danvers, MA, USA). MTT powder (1-(4,5-Dimethylthiazol-2-yl)-3,5-diphenylformazan) and METH powder were obtained from Sigma Chemical Co. (St. Louis, MO, USA). AnnexinV/PI apoptosis assay kit was purchased from BD Biosciences (San Diego, CA, USA). NE-PER Nuclear and Cytoplasmic Extraction Reagents Kit and ECL Western blotting detection reagents were obtained from Thermo Fisher Scientific (Waltham, MA, USA).

### 4.4. MTT Assay

Cellular viability was examined via MTT assay. SH-SY5y neuroblastoma cells (2 × 10^4^/200 μL) were incubated with indicated concentration (5–40 μM) of lupenone and/or 2 mM of METH for 24 h at 37 °C. The medium was discarded, and cells were treated with MTT (500 μg/mL) for 2 h. After incubation, cell culture plates were centrifuged, the supernatant was removed, and 150 μL of DMSO was added to each well to dissolve formazan crystals. These plates were read at 540 nm, and the absorbance of each sample was obtained relative to the control. The graph was generated with the percentage of control (% of control).

### 4.5. AnnexinV/PI Apoptosis Assay

Neuronal apoptosis of SH-SY5y cells was measured by a double staining method using annexinV conjugated with FITC and PI. Briefly, SH-SY5y neuroblastoma cells (2 × 10^5^) were treated with the indicated concentration of lupenone and/or 2 mM of METH for 24 h. Incubated cells were detached with 1× Trypsin-EDTA buffer and resuspended in 100 μL of 1× binding buffer (10 mM HEPES, 150 mM NaCl, 5 mM KCl, 5 mM MgCl_2_, 1.8 mM CaCl_2_) containing AnnexinV (20 μg/mL) and PI (1 μg/mL) for 15 min at RT. These cells were then evaluated on a BD FACSVerse (BD Biosciences, San Diego, CA, USA). The population of annexinV^+^ cells or annexinV^+^/PI^+^ cells was shown in the graph.

### 4.6. Western Blot Analysis

After incubation of SH-SY5y cells in desired condition, cells were lysed with a lysis buffer (1% Triton X-100, 150 mM NaCl, 20 mM Tris pH 7.5, one tablet of protease inhibitor, and one tablet of phosphatase inhibitor) for 30 min on ice and centrifuged at 14,000 rpm for 20 min at 4 °C. Approximately 40 μg of the lysate was loaded on 8–12% SDS–PAGE gels. After running, separated proteins were transferred onto PVDF membrane (Bio-Rad, Hercules, CA, USA). The membrane was blocked in 5% skim milk for 1 h, rinsed, and incubated with the indicated primary antibodies in 3% skim milk overnight. Excess primary antibodies were washed out in TBS with 0.1% of Tween 20 (TBS-T) four times and then incubated with 0.1 μg/mL peroxidase-labeled secondary antibodies (against rabbit or mouse) for 2 h. After three washes with TBS-T, bands were visualized with ECL Western blotting detection reagents (Thermo Fisher Scientific, Waltham, MA, USA) with ImageQuant LAS 4000 (GE Healthcare, Chicago, IL, USA). All observed bands were quantified with ImageJ software and normalized against a control.

### 4.7. NFκB Translocation Analysis by Western Blot

For NFκB translocation analysis, NE-PER Nuclear and Cytoplasmic Extraction Reagents (Thermo Fisher Scientific, Waltham, MA, USA) were used following the manufacturer’s instruction. Briefly, cells were lysed in CER buffer for 10 min on ice and centrifuged at 14,000 rpm for 5 min to separate cytosolic extracts from whole lysates. After centrifuge, supernatants were clearly removed, and pellets were lysed in NER buffer for 40 min on ice. After centrifuge, supernatants were collected as nuclear extracts. For confirmation accuracy of separation, anti-PARP and anti-β-actin antibodies were used respectively as a loading control. To detect the amount of p65 in cytosolic and nuclear extracts, a western blot was performed as described above.

### 4.8. Statistics

Mean values were calculated from the data obtained from at least three (usually three) separate experiments performed on separate days. Once the significance between multiple groups was determined by a one-way ANOVA test, Turkey’s test was used as post-hoc test to investigate the significance between individual groups. Differences between groups were considered significant at *p* < 0.05.

## Abbreviations

METH	methamphetamine
PD	Parkinson’s disease
PI3K	phosphoinositide 3-kinase
PI	propidium iodide
NFκB	nuclear factor kappa-light-chain-enhancer of activated B cells
IκB	inhibitor of kappa B
